# Camera-Integrable Wide-Bandwidth Antenna for Capsule Endoscope

**DOI:** 10.3390/s20010232

**Published:** 2019-12-31

**Authors:** Jong Jin Baek, Se Woong Kim, Youn Tae Kim

**Affiliations:** IT Fusion Technology Research Center and Department of IT Fusion Technology, Chosun University, Gwangju 61452, Korea; jongjinbaek0663@gmail.com (J.J.B.); swkim609@gmail.com (S.W.K.)

**Keywords:** meandered dipole antenna, capsule endoscopy, 2.4 GHz, industry–science–medical band, camera-integrable antenna

## Abstract

This paper presents a new antenna design for a capsule endoscope. The proposed antenna comprises a camera hole and meandered line. These features enable the antenna to be integrated on the same side as the camera, within the capsule endoscope. Moreover, light-emitting diodes can be mounted on the surface of the antenna for illumination. The antenna achieves a wide bandwidth, despite the small size owing to its meandered line structure.

## 1. Introduction

A capsule endoscope is an ingestible device used for the endoscopic examination of the digestive tract, which provides a painless and pleasant experience during the examination process. It comprises light-emitting diodes (LEDs), a camera, a wireless transmitter with an antenna, and a battery [1,2]. All of the components are located inside a small capsule that is externally coated with a biocompatible material in order to protect the digestive organs during capsule deployment and operation.

Various studies have been conducted to develop a suitably small antenna for capsule endoscopy [1,2,3,4,5,6]. The antennas in the literature [2,3,4,6] are located on the outside walls of the capsules. However, such conformal antennas necessitate a complicated manufacturing process of the antenna elements on the sidewall. Furthermore, the biocompatible-material coating layer can impact the performance of the antenna [3]. Unlike the conformal antenna, the modular ones in the literature [1,5] are located inside the capsule. The modular antenna is fabricated separately on a planar substrate, and no additional process is required during the capsule manufacturing. A dual camera scheme is required in order to reduce blind spots during the endoscopy procedure. To achieve this design, we used two cameras located at each end of the capsule [7].

As described in the literature [1,2], the modular antenna is located on top of the stacked modules, which comprise a camera, battery, and transceiver. The modular antenna is located opposite the camera module so as to enable the radiation of the electromagnetic field from the antenna without obstructing the camera’s view. However, it is possible to use dual cameras by placing another camera on the same side as the antenna.

A new antenna design for a capsule endoscope is proposed in this study. The proposed antenna is ring-shaped in order to enable the camera to be integrated on the same side of the capsule endoscope as the antenna. The antenna is designed to operate in the 2.4 GHz industrial, scientific, and medical (ISM) band. A meandered dipole structure is used as the radiating element to reduce the antenna size.

## 2. Antenna Structure

**Figure 1:**
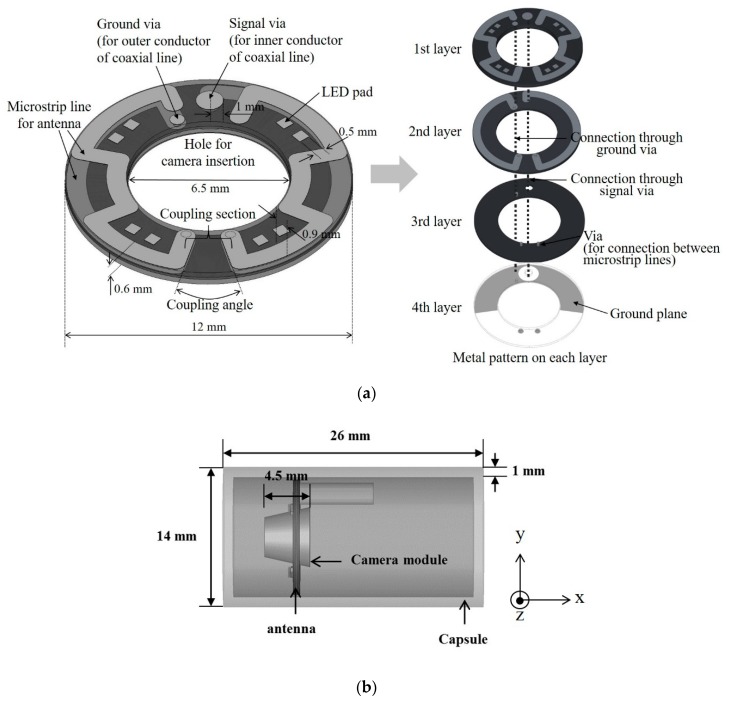
(**a**) Structure of the proposed antenna and (**b**) antenna placement within the endoscope capsule.

Figure 1a shows the structure of the proposed antenna and the layouts of each layer. The antenna structure is similar to that of a modified dipole antenna. The antenna substrate is composed of two Teflon layers, each having a 0.25 mm thickness, and are attached together using a pre-impregnated (prepreg) layer, whose electrical properties are similar to those of the Teflon layers. The microstrip lines for the field radiation are meandered on the first and second layers. As shown in Figure 1a, the microstrip lines on the second layer are connected to the lines on the first layer through the vias, so the microstrip lines for the field radiation end at the first layer. Similar to a conventional dipole antenna, the proposed antenna comprises two microstrip lines, each of which is connected to the signal and ground of an antenna feed line by the vias, as shown in Figure 1a. The signal via is connected to the right microstrip line on the second layer. A coaxial line is attached to the antenna for feeding. The inner connector of the coaxial line is inserted into the signal via and soldered. The ground via is connected to the left microstrip line on the second layer and to the ground plane on the fourth layer. The outer connector of the coaxial line is attached to the ground plane of the antenna by soldering; the ground plane is located on the fourth layer and is semicircular in shape. The ground plane distorts the polarization of the proposed antenna. The electric field needs to be coupled only between the two microstrip lines; however, the ground plane disturbs such field coupling. The ground plane is designed to not cover the entire bottom layer of the antenna, so as to improve the polarization property.

Generally, in capsule endoscopes, the antenna and camera have to be located on opposite sides of the capsule so as to prevent the antenna radiation from interfering with the camera recording. The proposed antenna offers a solution to this layout limitation by enabling the accommodation of both components on the same side. To this end, the structure of the proposed antenna has the following two features: a hole for camera insertion and a meandered line for securing LEDs. As shown in Figure 1a, the antenna substrate has a circular hole at the center, through which the capsule endoscope’s camera is inserted. After the assembly of all of the components, the camera is integrated at the center of the antenna facing outward. Owing to the camera hole, the antenna can be located on the same side as the camera, without obstructing the camera’s view. The microstrip lines on the first layer are meandered around the camera hole to effectively minimize the antenna size. Another advantage of the meandered line is that it provides space for the insertion of LEDs to illuminate the digestive tract during endoscope operation. The LEDs can be mounted in the empty spaces between the meandered lines. The metal pads for LED attachment are located next to the lines, as shown in Figure 1a.

**Figure 2:**
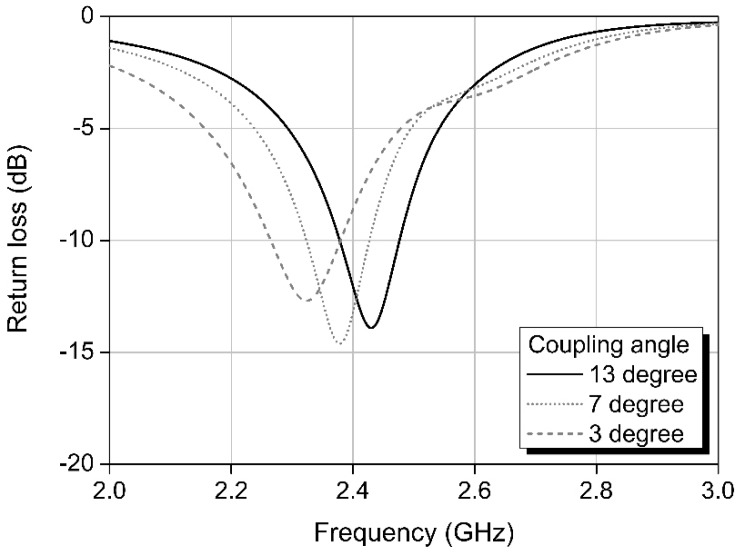
Simulation results showing return losses based on coupling angles.

The proposed antenna has a coupling section, in which the two transmission lines are closest to each other; it is used to control the length of the transmission line and, consequently, the center frequency of the antenna. In the coupling section, the feed signals transmitted through each line are coupled with one another. The signal coupling could affect the return loss of the antenna, so it is necessary to confirm a constant return loss, irrespective of the coupling area. Figure 2 shows the simulation results for the return losses for various coupling angles used to control the coupling area. CST Microwave studio software was used to simulate the return losses. In the simulation, Teflon layers with a dielectric constant of 2.17 were used as the antenna substrates. As can be seen from Figure 2, the center frequency decreases gradually as the coupling angle decreases. This is because decreasing the angle increases the length of transmission, and, consequently, decreases the resonant frequency of the antenna. The bandwidth, however, remains almost the same, irrespective of the coupling angle; hence, the coupling angle can control the center frequency of the antenna effectively without affecting the bandwidth.

From Figure 1b, it can be seen that the camera module is inserted into the antenna at the camera insertion hole. The camera module was modeled with a homogeneous dielectric layer with a dielectric constant of 4.2, which corresponds to the dielectric constant of polyvinyl chloride (PVC). Moreover, LEDs were attached to each of the metal pads, as shown in Figure 1a, after modeling with a semiconductor substrate with a dielectric constant of 10, which corresponds to the dielectric constant of silicon. The antenna gain was simulated after inserting the endoscope capsule in a human phantom with simulated muscle layers, an approach similar to that described in the literature [3]. In the simulation, the endoscope capsule was located in the middle of the human phantom, which was cubic in shape with sides of 12.5 cm in length. The maximum antenna gain was found to be approximately −29 dBi.

## 3. Antenna Fabrication and Performance Measurement

**Figure 3:**
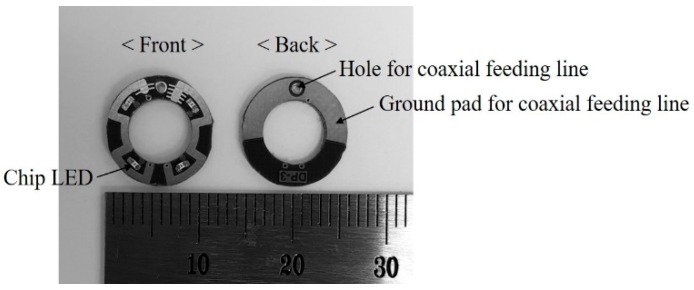
Fabricated antenna. LED—light-emitting diode.

Figure 3 shows the fabricated antenna, constructed from a Teflon substrate with a dielectric constant of 2.17. The LEDs for illumination can be seen to be mounted on the front. The ground pad was provided on the back surface so as to connect the antenna ground to the ground of the coaxial line used to feed the antenna.

**Figure 4:**
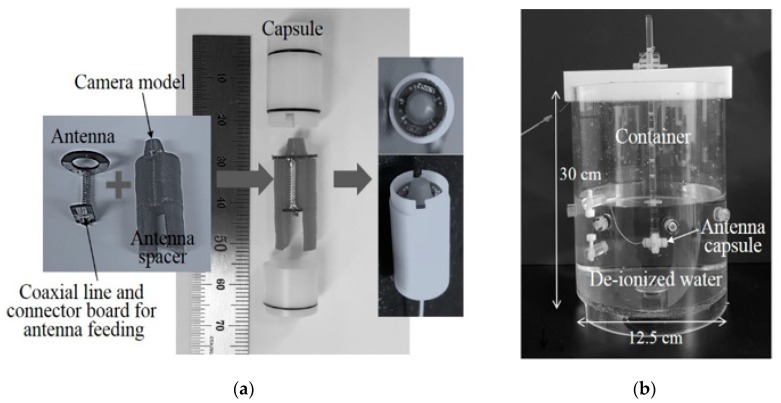
Measurement setup, namely: (**a**) antenna and camera model assembled within capsule; (**b**) capsule antenna inside tissue-simulating liquid.

The fabricated antenna was assembled within the capsule to measure the antenna performance, as shown in Figure 4a. The coaxial line and the connector board were soldered to the antenna, whereas the camera model was attached to the antenna spacer using glue. After assembling, the antenna was inserted into the antenna spacer. As integrating a camera with an antenna usually affects the antenna performance (as previously explained), the camera was modeled with a homogeneous dielectric material to prevent it from negatively impacting the antenna. The homogeneous material used to model the camera was PVC, whose dielectric constant is 4.2, considering that the capsule endoscope camera was made of plastic. The camera model was attached to the antenna spacer, which had a dielectric constant of less than 2.0. The fabricated antenna was connected to the coaxial line and connector board for the antenna feeding. After assembling the fabricated antenna and spacer, as shown in Figure 4a, the camera-integrated antenna was inserted within the polyacetal capsule. De-ionized water with a dielectric constant and conductivity of 56.91 and 97 μS/m, respectively, was used for the performance measurements in order to mimic the tissues of the human body. As a tissue-simulating liquid, de-ionized water is advantageous, because it contains fewer impurities, and the dielectric constant and conductivity are therefore highly reliable. Furthermore, the dielectric constant of de-ionized water is similar to that of muscle tissue at 2.4 GHz [8]. As shown in Figure 4b, the antenna capsule is placed inside a container filled with de-ionized water. The container is equipped with vertical and horizontal arms, which are used to fix the position of the antenna capsule.

**Figure 5:**
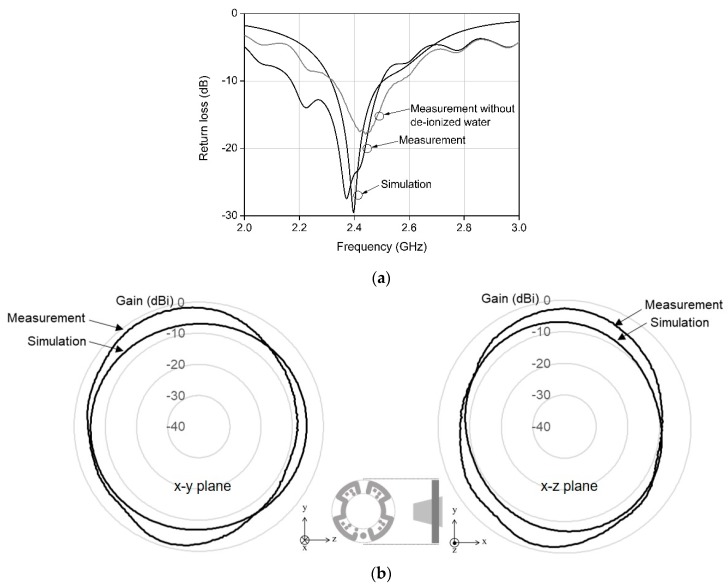
Measured and simulated results for (**a**) return losses and (**b**) antenna gains.

Owing to the mounting of LEDs onto the antenna surface and the integration of the camera model into the antenna, the return losses deviated from the simulated results. The antenna was tuned to compensate for such deviations. As the proposed antenna had a dipole structure, antenna tuning could be easily achieved by trimming the microstrip line on the first layer, as shown in Figure 1a. As previously described, the designed antenna had a structure similar to that of a modified dipole antenna; hence, the resonant frequency and resulting return loss could be controlled by trimming the microstrip line. For return loss optimization, measurements were conducted after placing the capsule inside the container, as shown in Figure 4b. The capsule was removed from the container and disassembled to shorten the microstrip line on the antenna surface. After the microstrip line was shortened, the capsule was assembled and subsequently placed in the container to measure the return loss. This procedure was repeated until the return loss was optimized to the center frequency. In the simulations, the camera and LEDs were modeled to obtain the return losses, while considering the deviations due to the camera model and LEDs. The modeling inaccuracy, however, caused an additional deviation of the return loss during measurement. Antenna tuning is thus necessary to compensate for the deviations caused by model inaccuracies. Figure 5a shows the measured return loss after antenna tuning, and the simulated signal loss when the antenna is placed in the de-ionized water. The measured signal loss has a wider bandwidth than the simulated signal loss, because the loss components of the capsule and the camera model could not be modeled in the simulation. Those loss components decreased the quality factor of the antenna during the measurement; consequently, the bandwidth was increased. The antenna achieved a wide bandwidth of over 330 MHz at 2.4 GHz. Owing to the effect of the LEDs and the camera on the return losses during the measurements, the simulated return loss was slightly different from the measured value. The simulated return loss in Figure 5a is also slightly different from that in Figure 2. This is because the antenna was located within the de-ionized water for the simulation results, as shown in Figure 5a, whereas it investigated the effect of the coupling section without the de-ionized water, as shown in Figure 2.

Figure 5a also shows the return loss measured after removing the de-ionized water from the container. The center frequency increased slightly, but the return loss remained low at 2.4 GHz. This means that the return loss does not affect the antenna gain, even when measured in free space. The antenna gain was measured in a full anechoic chamber. Figure 5b shows the antenna gain measured in free space at 2.4 GHz and the simulated gain. The measured average and maximum gains at 2.4 GHz were −4.9 and −1.3 dBi, respectively, on the x-y plane. The measured average gains showed small variations (of less than 3 dB) between 2.3 and 2.5 GHz. The measured gain in Figure 5b shows that the variation due to the elevation angle is less than 10 dBi; thus, the fabricated antenna is close to being isotropic. The gain was also simulated and measured on the perpendicular plane (i.e., the x-z plane), with no significant differences observed. This is because the antenna has meandered transmission lines on a circular substrate, and such symmetrical line distribution enables near-omnidirectional radiation patterns.

The measurement equipment used for the gain measurement did not support antenna rotation in the tissue-simulation liquid. Instead, the maximum gain was measured after antenna placement in the tissue-simulating liquid. Table 1 presents the measured maximum gain of the antenna designed in this study, along with a performance comparison with other antennas from a previous work. The conformal antennas from the literature [2,4,6] were large, because the antenna patterns were formed on the surfaces of the capsules. Owing to their large sizes, these antennas have relatively large bandwidths. The conformal antenna, however, requires a complicated manufacturing process, because antenna patterns must be formed on the vertical surfaces of the capsule sidewalls, causing difficulties in connecting the antenna outputs to modular transceivers, which are stacked with the other modules inside the capsule. The modular antenna offers an advantage in that the antenna output can be easily connected to the modular transceiver. Moreover, the fabrication of antenna patterns on vertical surfaces does not require a specific manufacturing process. Compared to the modular antenna in the literature [3], the proposed antenna has a larger bandwidth.

## 4. Conclusions

This paper presents the design of a small modular antenna for a capsule endoscope. The proposed antenna has a more efficient structure than conventional antennas, which enables the camera of the capsule endoscope to be integrated on the same side as the antenna. Owing to the meandered line structure, LED mounting can also be facilitated by the fabrication of metal pads between the lines. These features are distinctly advantageous in the application of the proposed antenna to capsule endoscopy.

## Figures and Tables

**Table 1:** Comparison of the antenna performance.

Antenna	Type	Size (mm)	Freq. (GHz)	BW (MHz)	Max.Gain (dBi)
[2]	Conformal	15.2 × 15	0.4	541	−17
[4]	Conformal	14 × 30	2.4	4300	−22.1
[6]	Conformal	12.3 × 30	1.4	300	−26
[3]	Modular	10 × 10	2.4	80	−32
This study	Modular	12 × 12	2.4	330	−29
